# Supplementary material for: Cholesterol sensing by CD81 is important for hepatitis C virus entry
Source: J Biol Chem. 2020 Sep 8;295(50):16931–48. doi: 10.1074/jbc.RA120.014761 (PMC7863897; doi:10.1074/jbc.RA120.014761)
Supplement: Supporting Information [file supp_295_50_16931__index.html]

Cholesterol sensing by CD81 is important for hepatitis C virus entry — CD81 cholesterol sensing — Cholesterol sensing by CD81 is important for hepatitis C virus entry — CD81 cholesterol sensing — Supporting Information 

# Cholesterol sensing by CD81 is important for hepatitis C virus entry

## Supporting Information

- Supplementary File 1 - Underlying data for Figure 6. Log2 LFQ intensity data and significance score is provided.
- Supplementary File 2 - Mass spectrometry protein identification data. Single peptide IDs are in italics. Potential contaminants, reversed sequences and those proteins only identified by site were filtered out and are shown in grey.
- Supporting Information (to be published online) - Supplementary Figures 1-6
